# PUBLIC HEALTH RESEARCHER PERSPECTIVES OF THE SUMMER RESEARCH INSTITUTE AND TRAINING NEEDS

**DOI:** 10.1093/geroni/igae098.1122

**Published:** 2024-12-31

**Authors:** Amy Thierry

**Affiliations:** Xavier University of Louisiana, New Orleans, Louisiana, United States

## Abstract

Over 30 public health early career researchers have participated in the Alzheimer’s Association Interdisciplinary Summer Research Institute (AA-ISRI) across the first three years of the program. During the week spent at AA-ISRI, public health participants attend a variety of sessions, including both full group sessions and track-specific sessions; receive mentoring from peers and expert faculty; and refine their research proposals. Public health track participants gain a greater understanding of the public health dimensions of dementia and its impact on communities and learn about the public health approaches to addressing dementia. The ultimate hope of the public health track of the AA-ISRI is that participants will depart having formed long-term connections and the tools needed to advance their research careers. During this presentation, AA-ISRI public health alum Dr. Amy Thierry will describe the experience of AA-ISRI as a participant, including the highlights and helpfulness of the didactic sessions offered at the program, the mentoring and networking opportunities that were available, and the impact that AA-ISRI had on the short-term long-term career trajectory and research. Since the AA-ISRI, Dr. Thierry has cultivated scholarly collaborations with members of her program cohort and has connected with AA-ISRI mentors and Alzheimer’s Association staff. Using the grant writing skills learned at the AA-ISRI, she has been awarded an NIH/NIA Loan Repayment Award for her project using Health and Retirement Study data to examine how neighborhood characteristics, structural stressors, and inflammation contribute to trajectories of cognitive function among racially/ethnically diverse US older adults across varying geographic contexts.

